# Hybrids of *Cinchona* Alkaloids and Bile Acids as Antiparasitic Agents Against *Trypanosoma cruzi*

**DOI:** 10.3390/molecules24173168

**Published:** 2019-08-30

**Authors:** Daniel Musikant, Aurélie Leverrier, Diana Bernal, Gabriel Ferri, Jorge A. Palermo, Martin M. Edreira

**Affiliations:** 1 Departamento de Química Biológica, Facultad de Ciencias Exactas y Naturales, Universidad de Buenos Aires, 1428 Ciudad deBuenos Aires, Argentina; 2 Departamento de Química Orgánica, Facultad de Ciencias Exactas y Naturales, Universidad de Buenos Aires, 1428 Ciudad de Buenos Aires, Argentina; 3 CONICET-Universidad de Buenos Aires, Unidad de Microanálisis y Métodos Físicos en Química Orgánica (UMYMFOR), 1428 Ciudad de Buenos Aires, Argentina; 4 CONICET-Universidad de Buenos Aires, Instituto de Química Biológica de la Facultad de Ciencias Exactas y Naturales (IQUIBICEN), 1428 Ciudad de Buenos Aires, Argentina; 5 Department of Pharmacology and Chemical Biology, School of Medicine, University of Pittsburgh, Pittsburgh, PA 15213, USA

**Keywords:** *Cinchona* alkaloids, bile acids, hybrids, *Trypanosoma cruzi*, amastigotes, antiparasitic activity

## Abstract

The current chemotherapy of Chagas disease needs to be urgently improved. With this aim, a series of 16 hybrids of *Cinchona* alkaloids and bile acids were prepared by functionalization at position C-2 of the quinoline nucleus by a radical attack of a norcholane substituent via a Barton–Zard decarboxylation reaction. The antitrypanosomal activity of the hybrids was tested on different stages and strains of *T. cruzi.* In particular, eight out of 16 hybrids presented an IC_50_ ≤1 μg/mL against trypomastigotes of the CL Brener strain and/or a selectivity index higher than 10. These promising hybrids yielded similar results when tested on trypomastigotes from the RA strain of *T. cruzi* (discrete typing unit—DTU—VI). Surprisingly, trypomastigotes of the Y strain (DTU II) were more resistant to benznidazole and to most of the hybrids than those of the CL Brener and RA strains. However, the peracetylated and non-acetylated forms of the cinchonine/chenodeoxycholic bile acid conjugate **4f** and **5f** were the most trypanocidal hybrids against Y strain trypomastigotes, with IC_50_ values of 0.5 and 0.65 μg/mL, respectively. More importantly, promising results were observed in invasion assays using the Y strain, where hybrids **5f** and **4f** induced a significant reduction in intracellular amastigotes and on the release of trypomastigotes from infected cells.

## 1. Introduction

The current pharmacological treatment (i.e., benznidazole (Bz) and nifurtimox (Nf)) against *Trypanosoma cruzi*, the etiological agent of Chagas disease, are highly toxic and not effective, especially during the chronic stage of the disease. In order to find alternative treatments against the disease, numerous studies have shown that quinoline derivatives display cytotoxic activity against different protozoan parasites [1,2,3,4,5,6]. A unique class of quinoline alkaloids are the *Cinchona cinchona* alkaloids, which includes quinine, quinidine, cinchonidine, and cinchonine. These naturally occurring compounds have all shown some degree of anti-parasitic activity, especially against *Plasmodium falciparum*. In particular, quinidine is the most active antiprotozoal alkaloid of this family and has been used for more than 400 years for the treatment of malaria [7].

With the strategy of combining the anti-parasitic properties of natural *Cinchona* alkaloids [8] with the known properties of bile acids as drug transporters [9], a series of 16 hybrids of *Cinchona* alkaloids and bile acids were prepared via a Barton–Zard decarboxylation reaction [10] (Table 1). Briefly, quinine, quinidine, cinchonine and cinchonidine were functionalized at position C-2 of the quinoline nucleus by a radical attack of a norcholane substituent. All the hybrids showed antiplasmodial activity (IC_50_ ≤ 6 μg/mL), particularly those containing a nor-chenodeoxycholane moiety (**4b**, **4d**, **4f**, **4h**, **5b**, **5d**, **5f**, **5h**) with IC_50_ values comparable to those of the natural alkaloids and selectivity indices in the range of 5.6–15.7 [10]. In addition, seven compounds (**4d**, **4f**, **4h**, **5b**, **5d**, **5f**, **5h**) showed promising trypanocidal activity against *T. brucei*, with IC_50_ values in the same range as the commercial drug suramin [10]. These results prompted us to evaluate the anti-trypanosomal activity of the hybrids against different strains and stages of *Trypanosoma cruzi.*

## 2. Results

A series of 16 hybrids of *Cinchona* alkaloids and bile acids were prepared via a Barton–Zard decarboxylation reaction, as previously described [10]. With the aim of evaluating the anti-trypanosomal differential activity of the hybrids on different stages and strains of *T. cruzi*, the hybrids were first tested on trypomastigotes of the reference strain of *T. cruzi*, the CL Brener strain. In parallel, cytotoxicity was assayed on NRK cells, a cell line that we have used as an infection model in the past [11]. To this end, the trypomastigotes and NRK cells were incubated with increasing concentrations of the hybrids and the calculated IC_50_ values (Table 2). All the hybrids showed some degree of trypanosomal activity. In particular, eight compounds—including the peracetylated and non-acetylated forms of the quinidine/litocholic bile acid conjugate (**4c** and **5c**), the cinchonine/chenodeoxycholic bile acid conjugate (**4f** and **5f**), and the cinchonidine/litocholic bile acid conjugate (**4g** and **5g**), as well as the peracetylated form of the cinchonidine/chenodeoxycholic bile acid conjugate (**4h**) and the non-acetylated form of the cinchonin/litocholic bile acid (**5e**)—displayed IC_50_ values below 1 μg/mL and/or selectivity indices of greater than 10 (Table 2, in grey).

Because of the high genetic variability and phenotypic diversity that *T. cruzi* presents, the parasite has been classified into six genetic groups (discrete typing units, DTUs) named TcI–TcVI [12]. The DTUs present different eco-epidemiological, clinical, and geographic associations, with several genetic molecular markers that are being used to classify the strains after their isolation from biological samples [12]. As a consequence of this variability, in vitro and in vivo differential drug susceptibility among strains has been reported [13,14,15,16,17]. Taking this into account, hybrids presenting an IC_50_ ≤ 1 μg/mL and/or a selectivity index (SI) ≥ 10 from the screening with CL Brener, were tested on trypomastigotes from the Y and the RA strains of *T. cruzi* (DTU II and VI, respectively) (Table 3). Trypomastigotes of the RA strain (DTU VI) showed a similar response to the treatment with the hybrids than those of the CL Brener strain, which is another member of the DTU VI. On the other hand, the Y strain (DTU II) was more resistant to the control drug, the commercial available Bz, and most of the assayed hybrids. However, the peracetylated and non-acetylated forms of the cinchonine/chenodeoxycholic bile acid conjugates **4f** and **5f** had IC_50_ values of 0.50 and 0.65 μg/mL, respectively, against Y strain trypomastigotes. It is noteworthy that these results represented a 20–30-fold difference compared to the IC_50_ value of Bz.

**Figure 1:**
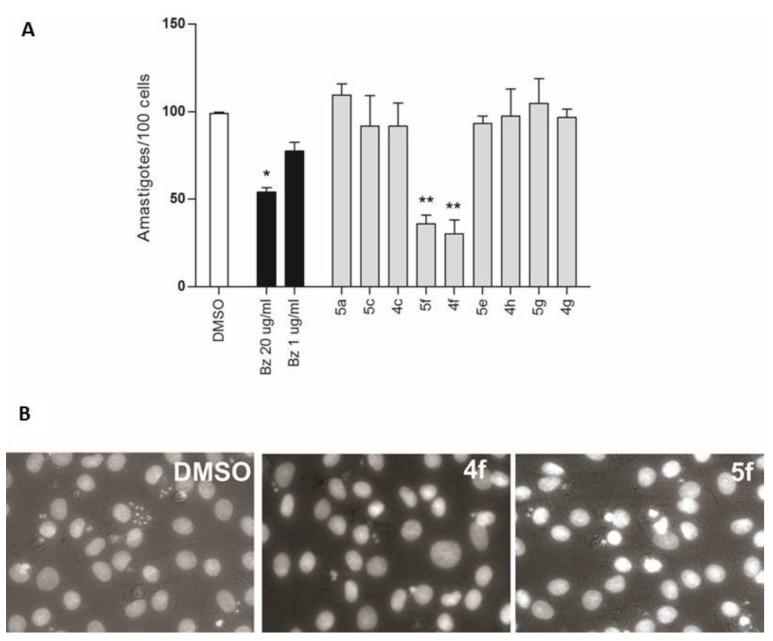
(**A**) Effects of hybrids on intracellular amastigotes. Hybrids were evaluated at the estimated parasite IC_50_ concentration for the Y strain (Table 3). Bars indicate mean ± SE of at least three independent assays (see Methods). * *p* < 0.05, ** *p* < 0.01. (**B**) Representative microscope photographs of NRK infected cells treated as indicated.

Not only the genetic diversity among strains should be taken into account while searching for new drugs. Differential susceptibility of the different life cycle stages of the parasite within the same strain [18] should be also considered. In this regard, Bz and Nf effectiveness against axenic epimastigotes and the intracellular stages of *T. cruzi* have been already reported [19]. Furthermore, drug sensitivity exhibited by the extracellular forms (i.e., epimastigotes and trypomastigotes) could sometimes be higher than the sensitivity of the intracellular form of the parasite, in part because of its intracellular availability [20] More importantly, given that in the chronic phase of Chagas disease, current chemotherapy is not efficient and that parasitemia is usually low, performing new drug screenings on the intracellular replicative stage of the parasite appears to be the better approach. To confirm their anti-parasitic activity, nine of the hybrids were evaluated against intracellular amastigotes of the Y strain at the IC_50_ found for trypomastigotes of the same strain. Briefly, trypomastigotes were incubated with NRK cells and left to infect for two hours. After the infection period, free trypomastigotes were removed from the medium, monolayers were washed, and media containing the final concentration of the drug were added. Forty-eight hours post infection, cells were fixed and stained, and amastigotes/100 cells were calculated. As shown in Figure 1A,B, a significant reduction in intracellular amastigotes was observed for the hybrids **4f** and **5f** compared to untreated infected cells. 

**Figure 2:**
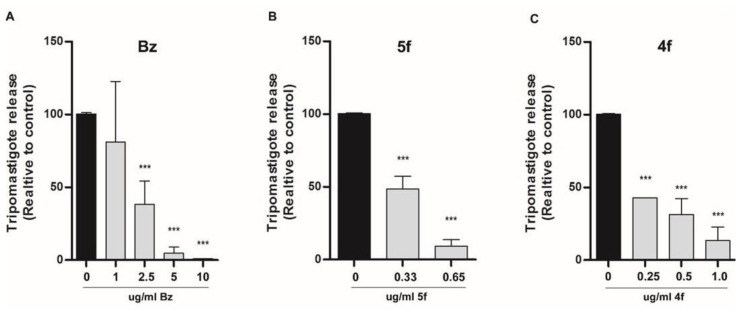
Hybrid activity on trypomastigote release at day six post infection. Cells treated with the indicated concentration of benznidazol (**A**) or the hybrids compounds **5f** (**B**) or **4f** (**C**). Bars indicate mean ± ES of at least three independent assays (see Methods) *** *p* < 0.001.

To fully eliminate the intracellular parasite, a trypanocidal drug action is ideally desired. For trypanostatic drugs, a longer chemotherapy is required to allow the elimination of the intracellular parasite, since the anti-parasitic effect could be reversed upon removal of the drug. In order to characterize the antiparasitic features of the hybrids, our strategy was to remove the hybrids from the medium of infected NRK cells and let the infection develop. In this approach, after the two hour infection of NRK cells with trypomastigotes of the Y strain, compounds were added and left for 72 hours before being replaced with fresh medium without drugs. Six days post infection, the trypomastigotes released to the supernatant were quantified. Two different scenarios were expected: 1) Upon removal of a trypanostatic hybrid, intracellular amastigotes would proliferate, and a higher trypomastigote release, close to control with no hybrid, would be observed; or 2) the hybrids would have a trypanocidal effect and non-viable amastigotes would not be able to proliferate, so the trypomastigote count would decrease. The results from Figure 2 clearly indicate that amastigotes could not recover from the 72 hours of treatment with hybrids **5f** and **4f**, since the trypomastigote count in the supernatant of infected cells was significantly lower than non-treated control. 

Overall, the results obtained with hybrids **4f** and **5f** are promising. These hybrids shown to be active against amastigotes of the Y strain, presenting a lower IC_50_ than Bz (Figure 1) and this anti-parasitic action could not reverse upon removal of the hybrids (Figure 2).

## 3. Discussion

An alternative approach to the discovery of new drugs to treat old neglected diseases, such as trypanosomiasis, could be the synthesis of new bioactive compounds through hybridization. In particular, the synthesis of hybrids of bioactive compounds that combine the properties of their individual components has emerged as a fast growing methodology in medicinal chemistry [5,10,21]. Following the strategy of combining the anti-parasitic properties of natural *Cinchona* alkaloids with the known properties of bile acids as drug transporters, a series of 16 hybrids of *Cinchona* alkaloids and bile acids were prepared via a Barton–Zard decarboxylation reaction. It was previously shown that these hybrids have anti-plasmodial and anti-trypanosomal activity [10]. In addition to these results, in this work, we have shown the promising trypanocidal activity of the hybrids against trypomastigotes of different DTUs of *T. cruzi*, such as CL Brener, RA, and Y. The high genetic variability and phenotypic diversity among strains of *T. cruzi* can lead to differential susceptibilities to drugs, suggesting that a broader screening, including different strains from different DTUs, should be performed in the search of new therapeutic drugs. In fact, we observed that the trypomastigotes of the Y strain were more resistant than the trypomastigotes of the CL Brener and RA strains to Bz and most of the newly synthetized hybrids. However, hybrids **4f** and **5f** presented a strong activity against trypomastigotes of the Y strain, as well. More importantly, a significant anti-parasite activity was found when these hybrids were tested on Y strain amastigotes, the intracellular proliferative stage of the parasite. This activity was reflected in a significant reduction in the number of intracellular amastigotes per infected cell. In addition, the action of hybrids **4f** and **5f** appeared to be trypanocidal, since amastigotes could not recover to proliferate and differentiate when the infection was left to develop. This fact that was reflected in a decreased in the number free trypomastigotes in the supernatant of infected cells after removal of the hybrids. 

## 4. Materials and Methods

### 4.1. Cells and Parasites

The NRK and Vero cell lines were routinely maintained in DMEM (Gibco) supplemented with 10% SFB (Natocor) and Penicillin/Streptomicin (100 Units/0.1mg/mL, Sigma) at 37 °C and 5% CO_2_ atmosphere. The trypomatigotes of *T. cruzi* strains CL Brener, Y, and RA were routinely maintained in Vero cells cultured in DMEM supplemented with 4% SFB and Penicillin/Streptomicin. The trypomastigotes of each strain were purified from infected Vero cells supernatants and used in the different assays.

### 4.2. Parasite IC_50_ Estimation

2 × 10^6^/mL trypomastigotes were incubated with different concentrations of the hybrids, control drug or vehicle, by triplicate at 37 °C and 5% CO_2_ for 24 h. Next, trypomastigotes were counted in a Neubauer chamber. The IC_50_ ± SD (n = 3) were estimated using the “Dose–Response” module in Graphpad Prism.

### 4.3. Cells IC_50_ Estimation

1 × 10^5^/mL NRK cells were grown overnight in a 96 multi-well plate. The culture medium was replaced by a culture medium containing increasing concentrations of hybrids, control drugs or vehicles, and cells incubated at 37 °C and 5% CO_2_ for 48 h. Next, cells were washed, fixed for 10 min with cold methanol (Sintorgan), and stained with violet crystal (Sigma 0.5% in methanol). After an exhaustive wash, cells were dried overnight. 10% acetic acid (Biopack) was added to each well, and the absorbance measured at 600 nm. Similarly, a standard curve was prepared (absorbance vs. increasing concentrations of NRK cells) to estimate the NRK IC_50_ using Graphpad Prism.

### 4.4. Amastigote Count

Infections were performed as previously described [11]. Briefly, NRK cells (growing on glass slides in a 24 multi-well plate) were infected for 2 h with trypomastigotes of the Y strain. After extensive washing, a medium containing compounds at the corresponding trypomastigote IC_50_ concentration (see Table 1) was added. After 48 h of incubation, cells were fixed with 4% formalin, stained with DAPI and photographed in a fluorescence microscope (Olympus). The amastigotes/100 cells were determined counting 1000 cells from each well (3000 cells/compound) using the ImageJ cell counter plugin. 

### 4.5. Trypomastigote Release Assay

NRK cells were cultured and treated as described in Section 4.1. At day 3 post infection (pi), the medium with hybrids was replaced by fresh media (with 4% SFB). At day 6 pi, the released trypomastigotes in the supernatant of infected were counted using a Neubauer chamber. 

### 4.6. Statistics

In all cases, 3 independent experiments were done by triplicate. In amastigotes count and trypomastigotes released from infected cells, the results are presented as normalized relative to control without drug. The mean ± SD of amastigotes/100 cells or trypomastigote released were calculated and analyzed with one-way ANOVA with Dunnett posttest performed with Graphpad Prism software.

## Figures and Tables

**Table 1:** Hybrids of *Cinchona* alkaloids and bile acids were prepared via a Barton–Zard decarboxylation reaction.

Hybrid (Alkaloyd + Bile Acid)	Peracetylated	Non-Acetylated
Quinine + Litocholic	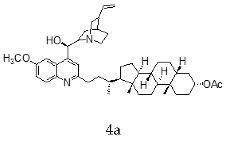	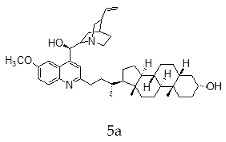
Quinine + Chenodeoxycholic	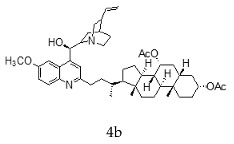	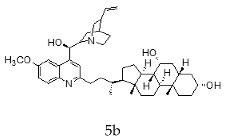
Quinidine + Litocholic	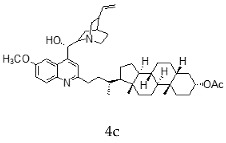	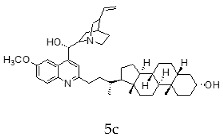
Quinidine + Chenodeoxycholic	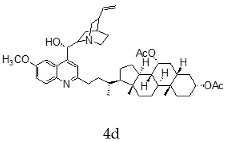	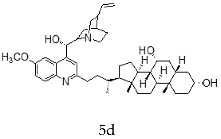
Cinchonine + Litocholic	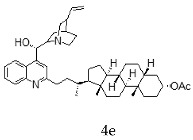	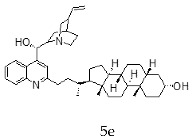
Cinchonine + Chenodeoxycholic	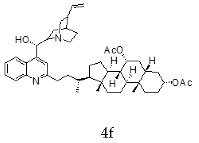	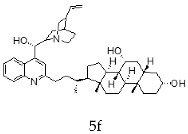
Cinchonidine + Litocholic	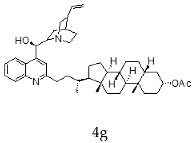	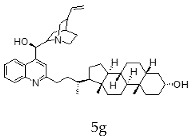
Cinchonidine + Chenodeoxycholic	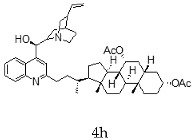	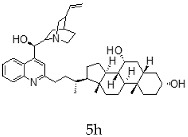

**Table 2:** IC_50_ values for trypomastigotes and NRK cells were determined as described in Materials and Methods. The selectivity index (SI) was calculated as IC_50_NRK/IC_50_ parasite. Hybrids with an IC_50_ close to 1 μg/mL and/or a selectivity higher then 10 (in grey) were selected as cytotoxic studies using other strains and for infection assays (see text). ND: Not Determined.

	*T. cruzi* CL Brener	NRK Cells	Selectivity
Hybrid	IC_50_ (μg/mL)	IC_50_ (μg/mL)	NRK/*T. cruzi*
5b	0.90 ± 0.10	5.10 ± 0.76	5.67
4b	0.80 ± 0.13	3.91 ± 0.29	4.89
5a	3.71 ± 0.13	15.30 ± 4.04	4.12
4a	0.64 ± 0.15	1.41 ± 0.13	2.20
5d	0.40 ± 0.05	1.59 ± 0.54	3.98
4d	0.72 ± 0.07	3.30 ± 0.08	4.58
5c	0.78 ± 0.11	>11	>10
4c	1.08 ± 0.23	16.53 ± 0.18	15.30
5f	0.34 ± 0.03	4.02 ± 0.55	12.18
4f	0.51 ± 0.06	6.69 ± 1.51	13.04
5e	3.96 ± 2.69	>30	ND
4e	1.16 ± 0.15	7.50 ± 0.29	6.46
5h	0.30 ± 0.00	0.67 ± 0.05	2.23
4h	0.70 ± 0.18	6.50 ± 0.28	9.28
5g	2.56 ± 0.58	27.11 ± 4.54	10.58
4g	1.30 ± 0.01	12.30 ± 0.81	9.46
Bz	2.5 ± 0.01	ND	ND

**Table 3:** Hybrid IC_50_ values for *T. cruzi* tripomastigote strains from different discrete typing units (DTUs).

	*T. cruzi* RA (DTU VI)	*T. cruzi* Y (DTU II)
Hybrid	IC_50_ (μg/mL)	IC_50_ (μg/mL)
5c	0.79 ± 0.19	≥2.00
4c	ND	≥1.00
**5f**	0.31 ± 0.10	0.65 ± 0.07
**4f**	0.25 ± 0.10	0.50 ± 0.03
4h	1.50 ± 0.50	≥2.00
5g	3.03 ± 0.24	3.25 ± 0.67
4g	1.10 ± 0.21	3.05 ± 0.71
Bz	2.5 ± 0.32	≥15.00
